# How to avert a hidden trap: the severe obturator nerve reflex

**DOI:** 10.1590/S1677-5538.IBJU.2020.0568.1

**Published:** 2021-03-25

**Authors:** Diego Moreira Capibaribe, Luciana Saboya Brito Dal Col, Mehrsa Jalalizadeh, Leonardo O. Reis

**Affiliations:** 1 Pontifícia Universidade Católica de Campinas Divisão de Oncologia Urológica e Laboratório de UroSciências CampinasSP Brasil Divisão de Oncologia Urológica e Laboratório de UroSciências, Pontifícia Universidade Católica de Campinas - PUC-Campinas, Campinas, SP, Brasil;; 2 Universidade Estadual de Campinas CampinasSP Brasil Universidade Estadual de Campinas - Unicamp, Campinas, SP, Brasil

## COMMENT

The obturator nerve is a mixed one, carrying motor and sensory fibers. It arises from the anterior primary rami of L2, L3, and L4 lumbar plexus and runs close to the bladder wall on its inferior/lateral portion, and exits the pelvis below the superior pubic rams, through the obturator canal, entering the adductor region of the thigh (1, 2).

Few nerves this size reach so much importance in one specific medical specialty as the obturator nerve. It plays a dramatic role in radical prostatectomies, pelvic lymphadenectomies and transurethral resections in one way or another and urologists have been trying to find ways to overcome the reflex caused by the obturator nerve stimulation of the adductor muscles of the leg when performing endoscopic resections of the bladder in lateral tumors for many years.

The prevention of the obturator's “jerk” or reflex comprises many aspects. A 2018 review described surgical (i.e. reducing diathermy current and bladder volume and intermittent or a staccato resection) and anesthetic techniques that can be executed to prevent this from happening (1) and in return, the surgeon may execute the resection thoroughly, without perforations and with more accuracy.

Anesthetic techniques involve neuromuscular blockade and selective blockade of the obturator nerve. While neuromuscular blockade involves general anesthesia, intubation, and many other aspects that make this choice more morbid, the selective blockade can be performed rather easily and with low rates of complications (3).

First described in 1922 by Labat (4), the obturator nerve blockade (ONB) originally depended on multiple punctures and paresthesia feeling for its localization before the advent of nerve stimulation and ultrasound-guided approach (5). Wassef (6) described the inter-adductor approach in 1993 and Pladzyk et al. demonstrated the efficacy and safety (complication <2%) in a study where 542 ONB were performed with 94% of success (3).

Bolat et al. pioneering brought up an interesting discussion regarding which form of energy is safer concerning significant obturator nerve reflex and consequent bladder perforation in patients under obturator nerve blockade (ONB) (7). The main challenge is related to the tiny room for improvement once severe obturator jerk occurrence on ONB is <3% (8). Which means that such infrequent and variable event will need thousands of patients randomized to find a difference between groups and techniques studied. Therefore, the number of patients in the study, although restricted and very well selected, has reduced statistical importance. Among 80 patients under ONB randomized 1:1 to monopolar and bipolar energy, there was no bladder perforation, obturator jerk was detected in 2 patients only and the article does not clarify whether the 3 patients who presented severe complications (Clavien >3) presented obturator jerk (7).

Unrelatedly to ONB, two metanalyses showed that bipolar resection, when compared to monopolar energy, tend to be more efficacious and safer, presenting better hemostasis, clearer working field that allows for better resection, muscle sampling, under lower temperatures with a trend for fewer artifacts (9, 10); however, more robust randomized controlled studies are needed.

TURBT is the foundation for decision making in BC and one of the first endoscopic procedures that trainees can perform, beyond best practices to optimize all aspects of TURBT, development of new instruments and techniques are warranted (11). The miniaturization and gastrointestinal endoscopy and laparoscopic surgery transference of technologies might contribute to the “endovesical surgery” in the future as a unique opportunity to the new surgeon-scientist generation to improve patient care.
